# A retrospective evaluation of the BIOFIRE FilmArray Blood Culture Identification 2 panel for the detection of pathogenic yeasts

**DOI:** 10.1128/spectrum.02258-25

**Published:** 2025-10-22

**Authors:** Emily Puumala, Noelle H. Adler, Sharon M. Deml, Nancy L. Wengenack

**Affiliations:** 1 Division of Clinical Microbiology, Department of Laboratory Medicine and Pathology, Mayo Clinic 6915, Rochester, Minnesota, USA; Montefiore Medical Center and Albert Einstein College of Medicine, Bronx, New York, USA

**Keywords:** clinical diagnostics, blood culture, yeast, syndromic panels

## Abstract

Bloodstream infections (BSIs) caused by opportunistic yeasts, namely those of the genus *Candida*, demonstrate strikingly high mortality rates and require early therapeutic intervention to optimize patient outcomes. Advancements in microbiological diagnosis of BSIs have introduced rapid molecular methods, including multiplex PCR panels, to detect common organisms directly from blood culture media. The BIOFIRE FilmArray Blood Culture Identification 2 (BCID2) assay is one such method and detects seven of the most common yeast species responsible for fungemia, enabling timelier therapeutic optimization by clinicians. Here, we evaluated the clinical performance of BCID2 for yeast identification from positive blood cultures between 2021 and 2025 at a tertiary medical center, compared to the reference standard method, which couples subculturing and diagnostic mass spectrometry. A total of 252 individual yeast identification events were confirmed using the reference method during our study period, 33 (13%) of which represent organisms that lack BCID2 PCR-based targets and 219 (87%) represent organisms targeted by this assay. Of the 219 identifications compatible with the BCID2 panel targets, 209 (95%) were correctly identified. Discordance between BCID2 and the reference method commonly stemmed from the inability of BCID2 to target all yeasts encountered clinically in blood culture, as well as its reduced accuracy in cultures where >1 yeast is present. Overall, these data suggest that the BCID2 assay is a rapid and accurate method to identify the most common organisms causing yeast BSI from blood culture; however, subculture-based reference techniques are important complementary tools in the clinical microbiology laboratory.

**IMPORTANCE:**

Invasive fungal diseases, including bloodstream infections (BSIs) caused by yeasts, are increasing in prevalence alongside the growing global population of immunocompromised individuals. Prompt and targeted treatment is necessary for effective management of yeast BSI, and microbiological diagnostics play an important role in tailoring patient treatment. Rapid molecular assays that simultaneously detect multiple pathogens associated with a specific syndrome are attractive strategies to expedite pathogen identification. This includes the BIOFIRE FilmArray Blood Culture Identification 2 (BCID2) assay, which detects 33 organisms including 7 yeasts commonly associated with fungemia, directly from blood culture media. Its performance has been well established for the detection of bacteria and bacterial resistance markers from blood cultures. Here, we provide a yeast-specific snapshot into the microbiological performance of BCID2, highlighting its accuracy as well as limitations in detecting medically important yeasts compared to standard culture-based identification from a four-year specimen cohort collected in a tertiary care hospital.

## OBSERVATION

Yeasts, namely those of the genus *Candida,* are major contributors to the growing global burden of fungal infection. Recent estimates suggest that the annual incidence of invasive candidiasis and *Candida* bloodstream infection (BSI) sits at over 1.5 million cases with a mortality rate of greater than 60% (1). Rapid and appropriate treatment of *Candida* BSI upon clinical suspicion, particularly in high-risk populations, has a substantial impact on patient outcomes (2). Timely and accurate diagnosis also plays a crucial role in the effective management of these infections. Blood cultures remain the gold-standard methodology for the identification of yeasts in the context of BSI, and when coupled with identification using conventional methods, such as matrix-assisted laser desorption ionization-time of flight mass spectrometry (MALDI-ToF MS), they demonstrate exceptional accuracy in the identification of most pathogenic yeast species (3).

Rapid molecular diagnostic assays that detect multiple pathogenic targets of a common syndrome have emerged as attractive tools to automate and expedite pathogen identification. The multiplex PCR-based assay, BIOFIRE FilmArray Blood Culture Identification 2 (BCID2; bioMérieux, Inc, Salt Lake City, USA), directly tests positive blood culture media for a panel of 43 molecular targets, including markers for 26 bacteria, 7 yeasts, and 10 antimicrobial resistance genes, yielding results in approximately 1 h (4). This panel has demonstrated high levels of sensitivity and specificity and can assist clinicians in rapidly refining their treatment plans (5–7). Among the yeasts targeted by the BCID2, there are five *Candida* species that account for >90% of invasive human *Candida* infections: *C. albicans, C. tropicalis, C. glabrata, C. parapsilosis*, and *C. krusei*. Additionally, *C. auris* (*Candidozyma auris*) and *Cryptococcus neoformans/gattii* are targeted and represent organisms in the Critical Priority Group of the World Health Organization’s Fungal Priority Pathogens List (8). The diverse antifungal susceptibility profiles exhibited by common yeasts highlight the importance of genus and further species-level identification in cases of BSI (2). For example, while *C. albicans* is routinely susceptible to antifungals spanning the azole, echinocandin, and polyene classes, up to 97% of *C. krusei* isolates demonstrate resistance to the first-generation azole, fluconazole (9).

Although several studies have been conducted to evaluate the performance of BCID2 in the clinical laboratory setting, there is a dearth of yeast-focused data sets to highlight the assay’s specific utility in the detection of fungemia. Here, we aimed to retrospectively evaluate the performance of the BCID2 assay in comparison to culture coupled with MALDI-ToF MS (Bruker Daltonics, Bremen, Germany), which was used as the reference method for identification of medically important yeasts in blood culture. A data set of all blood culture orders that generated a positive result for yeast and were tested using the BCID2 assay between its implementation at our institution in June 2021 and February 2025 was compiled. Data were generated from specimens collected at Mayo Clinic Rochester, Minnesota, a >2,000-bed tertiary care hospital, revealing 255 individual yeast identification events from first-time positive blood culture bottles and a total of 252 yeast isolates. These data are not inclusive of blood cultures that were repeatedly positive for the same patient on a single admission. The laboratory workflow employed for workup of patient blood cultures generally includes initial incubation of two Plus Aerobic/F and one Lytic/10 Anaerobic/F BD BACTEC (Beckton, Dickinson and Company, Sparks, USA) blood culture bottles, which are inoculated per standard adult culture collection. The aerobic and anaerobic BD BACTEC culture bottles are effective in detecting bacteria, *Candida*, and other yeasts (10). Upon positivity, bottles are removed from incubation, and a Gram stain is performed on the positive media to inform combinatorial follow-up testing. Media from the first positive blood culture bottle from a given collection that demonstrates organisms consistent with bacteria or yeast on Gram stain are forwarded to the BCID2 panel for rapid identification. Media from all blood culture bottles demonstrating yeast-like morphologies on Gram stain, regardless of time to positivity within a given collection set, are reflexed to a calcofluor white/potassium hydroxide fungal stain and subcultured on Sabouraud’s dextrose agar or inhibitory mold agar for isolation and confirmatory MALDI-ToF MS identification. Microscopy and BCID2 assay results are released as preliminary reports to the patient’s electronic medical record, and confirmatory culture and MALDI-ToF MS identifications inform the final report. We note that an analogous bacterial culture workup is performed for blood cultures staining positive for bacteria; however, we have elected to focus on the yeast identification process for the purpose of this observation.

The 4 year study encompassed 255 individual yeast identification events from first-time positive blood cultures, and resulted in the BCID2 and the reference method yielding concordant results for 209 (82%) identifications, and discordant results in 46 (18%) identifications (Table 1). Initial yeast identifications by Gram stain and BCID2 were predominantly made from aerobic blood culture bottles (84%), consistent with findings in the literature that yeast detection rates from BD BACTEC aerobic plates are generally higher (11). The frequency of discordant results was similar regardless of whether the BCID2 was performed on aerobic or anaerobic culture media at 18% and 17.5%, respectively. Discordant results were classified as false positives, false negatives, or incorrect identifications. False positives were defined as yeast identifications made by the BCID2 panel, which were followed up by a culture negative for yeast. False negatives indicate BCID2 results returning as “yeast not detected,” where stains and/or cultures confirmed the presence of yeast from the patient collection in question. Incorrect identifications constitute BCID2 results that indicated the presence of yeast; however, the identification did not match that of the reference method. As blood culture bottles are screened for positivity by stain, prior to performing BCID2, there were no false positives identified across our cohort; however, 36 BCID2 false negatives were identified, comprising 78% of all discordant identifications. Of these false negatives, 83% (*n* = 30) represented yeasts that the BCID2 panel does not target; therefore, these results were as expected (Tables 1 and 2). For the remaining 17% (*n* = 6), a review of the culture workups revealed that all six arose from cultures containing both bacteria and yeast. In these six cases, yeast identified by the reference method was isolated from a culture bottle that became positive following the initial positive bottle (containing bacteria only) within a common three-bottle collection. Per our current workflow, only the bottle that turns positive first, regardless of morphologies seen on Gram stain of subsequently positive bottles, is tested using BCID2. We also confirmed that there were no instances in the study period where blood culture bottles demonstrated bacteria only on Gram stain, while BCID2 subsequently detected yeast. Overall, the six cases in question may not represent true false negatives. They do, however, represent discordance between the BCID2 and reference method used in our laboratory for identification of yeast from blood culture and reflect how the implications of workflow-related measures may influence sensitivity of pathogen detection from blood culture.

**TABLE 1:** Summary of overall agreement between the BIOFIRE FilmArray BCID2 panel and reference culture-based identification method for yeasts isolated from blood culture bottles between 2021 and 2025

BCID2 yeast result agreement with the reference method	Total
Concordant identifications	209
Discordant identifications	46
Yeast identified by BCID2 but not the reference method	0
Yeast not targeted by BCID2	30
Yeast targeted by BCID2 but not detected in first bottle	6^*a*^
Incorrect identifications	10
*≥1 organism(s) in mixed culture identified but not present***^*b*^**	3
*≥1 organism(s) in mixed culture not identified*^*c*^	4
*Single organism misidentified*	3
Total yeast identifications from blood culture	255

^*a*^ The cases listed here were initially identified as BCID2 false negatives. Investigation determined that 6/6 represent cultures with both bacteria and yeast; however, the initially positive bottle run on BCID2 did not stain for or grow yeast. Ultimately, yeast was grown in subsequently positive bottles from the same collection. These data represent discordance between the BCID2 and reference method used in our laboratory for identification of yeast from blood culture and reflect how culture procedures influence sensitivity of pathogen detection from blood culture.

^*b*^ *C. tropicalis* represents the only organism identified as a second yeast organism by BCID2 but was not present per the reference culture method. In two instances, *C. parapsilosis* and *C. tropicalis* were identified by BCID2; however, only *C. parapsilosis* grew. In the remaining case, *C. glabrata* and *C. tropicalis* were identified by BCID2; however, only *C. glabrata* grew.

^*c*^ The yeasts identified in mixed cultures using the reference method, which were not identified by BCID2, included *C. albicans* (*n* = 3) and *C. glabrata* (*n* = 1). The unidentified *C. albicans* isolates were grown in mixed culture with correctly identified isolates of *C. glabrata* (*n* = 2) or *C. auris* (*n* = 1). The unidentified *C. glabrata* (*n* = 1) isolate was grown in mixed culture with correctly identified *C. albicans*.

**TABLE 2:** Summary of yeasts identified from blood cultures by the reference method (subculture and MALDI-ToF MS) between 2021 and 2025 with overall concordance of BCID2 results relative to the reference method of subculture followed by MALDI-ToF MS

BCID2 target	Species	Total isolates cultured	Identified by BCID2	Not identified by BCID2	Percent concordance (95% CI^*a*^)
Yes	*Candida albicans*	96	92	4	96 (89.4–98.7)
	*Candida* (*Candidozyma*) *auris*	1	1	0	100 (16.8–100)
	*Candida glabrata*	83	78	5	94 (86.3–97.7)
	*Candida krusei*	6	5	1	83 (41.8–98.9)
	*Candida parapsilosis*	22	22	0	100 (82.5–100)
	*Candida tropicalis*	5	5	0	100 (51.1–100)
	*Cryptococcus neoformans* complex	6	6	0	100 (55.7–100)
Total		219	209	10	95 (91.7–97.6)
No	*Candida bracarensis^c^*	1	0	1	0 (0–83.3)
	*Candida dubliniensis*	11	0	11	0 (0–30.0)
	*Candida guilliermondii* complex	3	0	3	0 (0–61.8)
	*Candida kefyr*	1	0	1	0 (0–83.3)
	*Candida lusitaniae*	6	0	6	0 (0–44.3)
	*Candida (Pichia) norvegensis*	1	0	1^*b*^	0 (0–83.3)
	*Candida orthopsilosis*	1	0	1	0 (0–83.3)
	*Candida pelliculosa (Wickerhamomyces anomalus*)	1	0	1	0 (0–83.3)
	*Malassezia pachydermatis*	2	0	2	0 (0–71.0)
	*Pichia manshurica*	1	0	1^*b*^	0 (0–83.3)
	*Rhodotorula mucilaginosa*	1	0	1	0 (0–83.3)
	*Saccharomyces cerevisiae*	3	0	3	0 (0–61.8)
	*Trichosporon* species	1	0	1	0 (0–83.3)
Total		33	0	33	0

^*a*^ 95% CI: 95% CI computed using the modified Wald method.

^*b*^ Both *Pichia* species identified following culture and MALDI-ToF MS were initially incorrectly identified as *C. krusei*.

^*c*^ Grey shaded values are yeast not included on the BCID2 panel.

A significant subset of the 10 incorrectly identified yeasts arose from blood cultures that ultimately grew >1 yeast organism on subculture (Table 1). We observed four cases where one yeast was identified by BCID2; however, an additional species was grown and identified using the reference method. For all instances, the organisms isolated were *Candida* sp. with BCID2 panel targets. Additionally, three cases were observed where *C. tropicalis* was identified in addition to a second *Candida* species by BCID2; however, it was not recovered upon subsequent cultures. These cases explain the discordance in our observation of 255 total yeast identification events by BCID2, while 252 individual yeasts were ultimately isolated using the reference method. In February 2024, BioFire Diagnostics, LLC issued a class II device recall on all non-expired BCID2 panel lots used in concert with BD BACTEC blood culture bottles due to trends in false identifications of *C. tropicalis* (12). It has been hypothesized that inviable *C. tropicalis* nucleic acids may be present in the BD BACTEC blood culture bottle media, leading to false identifications by BCID2 (12). Although the recall event is still classified as open, the three incorrect identifications of *C. tropicalis* in our data set were clustered between September 2023 and April 2024 (12). Further investigation of the presumptive and confirmed polymicrobial cultures identified in our laboratory revealed a total of five blood cultures that grew >1 yeast species, as verified by subculture and MALDI-ToF MS. All five grew combinations of *Candida* species with BCID2 targets using the reference method; however, the BCID2 assay only resulted in a complete identification of the yeasts present in one of the five events. This finding is in line with previous evaluations of the BCID2 assay in polymicrobial bacterial cultures and suggests that in our cohort, BCID2 was less sensitive than the reference method when identifying multiple yeasts grown in a single blood culture (13).

A further two incorrect identifications were the result of the BCID2 assay identifying *C. krusei* in cases where the reference method confirmed the presence of *Pichia manshurica* and *Candida* (*Pichia) norvegensis,* respectively. The BCID2 Instructions for Use indicates that the detection reagents used for *C. krusei* may cross-react with related species including *Pichia* species and *C. norvegensis* at positive blood culture levels, likely explaining the phenomenon we observed (4). The single remaining incorrectly identified blood culture was initially positive by BCID2 as *C. neoformans/gattii*; however, it grew *C. dubliniensis* using the reference method.

In examining the concordance between the reference method and BCID2, the data were stratified based on the organisms confirmed from each positive culture by species and their ability to be detected by the BCID2 panel (Table 2). Of the 252 unique yeast isolate identifications confirmed by culture and MALDI-ToF MS, 87% (*n* = 219) were targetable by the BCID2 assay. Considering this cohort in isolation, there was a 95% (*n* = 209) concordance between BCID2 and the reference method, which corresponds more closely with the published literature and manufacturer’s specifications (Table 2) (6, 14). Of the 13 species not targeted by BCID2, as anticipated, none were identified by the panel assay, highlighting its overall specificity. As previously discussed, the only organisms initially mischaracterized by BCID2 were *P. manshurica* and *C. norvegensis*, belonging to a genus with recognized cross-reactivity with the BCID2 *C. krusei*-targeted PCR.

Ultimately, our findings demonstrate that most yeast BSIs detected at Mayo Clinic Rochester between 2021 and 2025 were caused by the epidemiologically significant organisms included on the BCID2 panel. For >95% of yeast identifications corresponding to BCID2-detectable organisms (*C. albicans, C. glabrata, C. tropicalis, C. parapsilosis, C. krusei, C. auris,* and *C. neoformans/gattii*), the multiplex panel sensitively and accurately identified the yeast present in blood culture. Additionally, throughout our study period, the BCID2 assay method generated results an average of 2 days prior to the reference method in blood cultures positive for yeast, highlighting its power to provide clinicians with more timely organism identifications to drive therapeutic intervention. Still, coupling rapid, molecular blood culture identification methods like BCID2 with standard culture and identification techniques remained essential in nearly 15% of yeast-positive blood cultures, where organisms not targeted by the syndromic panel were observed. Additionally, in polymicrobial cultures where >1 yeast is present, despite our small sample size, the reference method was more sensitive in accurately identifying all yeast species present. This combinatorial approach provides an additional layer of quality control, ensuring that blood culture identifications returned by the BCID2 assay are confirmed by a secondary technique prior to finalizing a blood culture result. Ultimately, our findings provide a yeast-targeted snapshot into the performance of the BCID2 panel in a hospital setting since its implementation and highlight the power of rapid molecular testing modalities to quickly and accurately identify yeasts growing in blood culture, as well as the complementary role conventional culture and mass spectrometric methods continue to play in microbiological diagnosis of yeast BSI.
